# Passive physical barrier modulates UVB-induced METosis-related MPO expression and activity, 25-hydroxyvitamin D3-1alpha-hydroxylase, and the shift of tissue-resident macrophages toward M1-associated iNOS

**DOI:** 10.3389/fimmu.2025.1583493

**Published:** 2025-08-14

**Authors:** Farah Sara Meterfi, Souad Zoudji, Nour Elhouda Bendjeffel, Rabia Messali, Fadila Boudjelal, Chahrazed El Mezouar, Nawal Brikci Nigassa, Zineb Mekkaoui, Slimane Brikhou, Franck JD Mennechet, Chafia Touil-Boukoffa, Xin Li, Abdelouahab Bellou, Mourad Aribi

**Affiliations:** ^1^ Laboratory of Applied Molecular Biology and Immunology, University of Tlemcen, Tlemcen, Algeria; ^2^ Pediatrics Department, Mother and Child Specialized Hospital of Tlemcen, Tlemcen, Algeria; ^3^ Biochemistry Department, Tlemcen University Hospital Center, Tlemcen, Algeria; ^4^ Pathogenesis and Control of Chronic and Emerging Infections, The Institut National de la Santé et de la Recherche Médicale (INSERM) U1058, University of Montpellier, Etablissement Français du Sang, Antilles University, Montpellier, France; ^5^ Algerian Academy of Sciences and Technologies (AAST), El Madania, Algiers, Algeria; ^6^ Shenzhen Key Laboratory of Viral Oncology, The Clinical Innovation and Research Center (CIRC), Shenzhen Hospital, Southern Medical University, Shenzhen, China; ^7^ China-Algeria International Joint Laboratory on Emergency Medicine and Immunology, Guangdong Provincial, People’s Hospital (Guangdong Academy of Medical Sciences), Southern Medical University, Guangzhou, China; ^8^ Institute of Sciences in Emergency Medicine, Department of Emergency Medicine, Guangdong Provincial People’s Hospital (Guangdong Academy of Medical Sciences), Southern Medical University, Guangzhou, China; ^9^ Medical Research Institute, Guangdong Provincial People’s Hospital (Guangdong Academy of Medical Sciences), Southern Medical University, Guangzhou, China; ^10^ Department of Emergency Medicine, Wayne State University School of Medicine, Detroit, MI, United States; ^11^ Global Network on Emergency Medicine, Brookline, MA, United States; ^12^ Biotechnology Research Center (CRBt), Constantine, Algeria

**Keywords:** 25-hydroxyvitamin D3-1alpha-hydroxylase, physical barrier simulation, peritoneal tissue-resident macrophages, METosis-related MPO expression and activity, UVB exposure, M1 macrophage-associated iNOS activity

## Abstract

**Background:**

This study investigated the role of UVB radiation and the influence of a simulated passive barrier on the enzymatic conversion of 25-hydroxyvitamin D3 (25(OH)D_3_) by 1-alpha hydroxylase and its effects on the functional activity of tissue-resident macrophages.

**Methods:**

Murine peritoneal tissue-resident macrophages (PRMφs) were exposed to three conditions: (1) Baseline (Control group), with no light exposure; (2) UVB+/RF- group, exposed to UVB rays without passive barrier simulation; (3) UVB+/RF+ group, UVB exposure with a thin layer of rat fur to mimic the passive barrier on the skin.

**Results:**

UVB exposure did not significantly alter 25OHD_3_ levels across groups but led to a marked downregulation of 1-alpha hydroxylase, particularly with the simulated barrier. UVB slightly enhanced phagocytosis and significantly increased nitric oxide (NO) and hydrogen peroxide (H_2_O_2_) production. Moreover, hypochlorous acid (HOCl) levels were significantly upregulated in the UVB-exposed PRMφ group, whereas they returned to baseline levels in the UVB+/RF+ group. Furthermore, both MPO expression and activity were markedly upregulated after UVB exposure and downregulated in UVB+/RF+ group, suggesting that the overall effect of UVB on METosis-related MPO activity was substantially attenuated by the simulated barrier (for both comparisons, *p* < 0.001 by ANOVA test). Additionally, UVB exposure shifted PRMφs toward M1-phenotype, as evidenced by decreased ARG1 activity and increased iNOS activity and M1_(iNOS)_-to-M2_(ARG1)_ ratio. Additionally, UVB downregulated catalase (CAT) activity and intracellular glucose (_i_GLU) levels, with a stronger effect in the barrier group. While UVB increased total cellular cholesterol content (_tcc_CHOL), this effect was mitigated by the barrier. Finally, intracellular free calcium ion (_if_Ca^2+^) levels remained unaffected by UVB but showed a slight increase with the barrier.

**Conclusions:**

UVB exposure enhances tissue-resident macrophage function in a preclinical rat model, increasing respiratory burst, phagocytosis, and M1-like polarization. The simulated barrier modulates these effects, notably by reducing MPO expression and METosis-related activity, which suggests a potential attenuation of excessive inflammation. These findings provide valuable insights relevant to human immune modulation and support further translational research. Future studies should investigate the role of circadian rhythms and other cell types in UVB- and vitamin D-mediated immune modulation.

## 1 Introduction

Vitamin D (VD), recognized both as a vitamin and pre-hormone, assumes a crucial role in diverse physiological processes. Its active form, 1,25-dihydroxyvitamin D_3_ (1,25(OH)_2_D_3_), is integral for bone health and calcium balance (1, 2). Although conventionally linked to calcium metabolism and skeletal well-being, recent studies have uncovered its role as an immunomodulator (3).Various immune cells, including macrophages (4), express the vitamin D receptor (VDR), a nuclear receptor and ligand-activated transcription factor belonging to the superfamily of nuclear receptors (5). Notably, macrophages not only respond to vitamin D but also possess the ability to produce bioactive vitamin D (6).

The circulating form of vitamin D, 25-hydroxyvitamin D (25[OH]D), is predominantly bound to the vitamin D-binding protein (DBP) in the bloodstream, while 10–15% is bound to albumin, and less than 1% of circulating vitamin D exists in an unbound form (7). This binding stabilizes 25(OH)D, prolongs its half-life, and facilitates its delivery to target cells. The DBP25(OH)D complex interacts with specific receptors on the cell surface, such as megalin and cubilin, enabling endocytosis and internalization of the complex (8, 9). Inside the cell, 1,25(OH)_2_D_3_ binds to the VDR, located in either the cytosol or the nucleus. The activated VDR then forms a heterodimer with the retinoid-X receptor (RXR), which binds to DNA, thereby stimulating the production of antibiotic peptides like cathelicidin and β-defensin (10). Moreover, it is important to note that, unlike other steroid hormones in the body, which are synthesized directly from cholesterol, vitamin D synthesis requires both the 7-dehydrocholesterol precursor and UVB rays (290–320 nm) (11). In the absence of this reaction, humans must rely on dietary vitamin D intake, available in two forms: vitamin D_2_ (ergocalciferol) and vitamin D_3_ (cholecalciferol) (12, 13).

UVB at the Earth’s surface is influenced by various physical and temporal factors, including latitude, altitude (14), season (12), and weather conditions. Biological factors, such as skin melanin (13), and personal, cultural, and behavioral factors including clothing (15), holiday habits (16), and sunscreen use (17) as well as the extent of exposed body surface area (18), also affect the efficiency of UVB penetration, its interaction with skin cells for vitamin D synthesis, and the subsequent bioconversion process.

Moreover, it is of great importance to note that based on association studies, it has been reported that vitamin D intake, as well as circulating levels of 25(OH)D, are not systematically altered in certain pathological conditions, including autoimmune diseases, allergies, and other immune-related disorders. Conversely, insufficient vitamin D intake or reduced circulating levels have been detected in individuals who appear to be in good health. These observations emphasize that circulating 25(OH)D levels, although commonly used as standard indicators, may not be sufficient to fully assess the status of vitamin D and its role in both pathological and healthy contexts. These variations suggest that additional factors, such as differences in sun exposure, diet, or genetic capacity to metabolize vitamin D, as well as the possible presence of compensatory mechanisms, may influence the utilization and metabolism of vitamin D, including its conversion into the bioactive form, *i.e.*, 1,25(OH)_2_D_3_. It is likely that these compensatory mechanisms function normally in healthy conditions but may be altered in pathological contexts.

All these observations highlight the need to study the ‘active reservoir’ form of vitamin D, which is ready to be utilized (when necessary) in response to external stimuli, such as activation by inflammatory factors, rather than focusing exclusively on its circulating levels. This vitamin D form could correspond to ‘non-circulating vitamin D (tissue 25(OH)D_3_ levels)’, produced locally in keratinocytes under the influence of UVB rays and directly used by local immune cells, such as tissue-resident macrophages. In this way, it would play an essential and direct role in modulating the immune response, compensating for the effect of circulating vitamin D deficiency. This form is not found in the bloodstream, but could be crucial for the local functions of immune cells. Here, we hypothesize that tissue-resident macrophages could be influenced by UVB exposure. This hypothesis is based on two aspects: first, their capacity to reprogram their cholesterol metabolism upon activation and exposure to inflammatory signals (19); and second, their involvement in the synthesis of bioactive molecules, including vitamin D metabolites, serving as a source of extrarenal production of 1,25(OH)_2_D_3_ (20). Based on the aforementioned, this pioneering study aims to evaluate the impact of UVB radiation and passive physical barrier on both the intracellular conversion of 25(OH)D_3_ and the overall functional activities of tissue-resident macrophages.

## 2 Materials and methods

**Figure 1:**
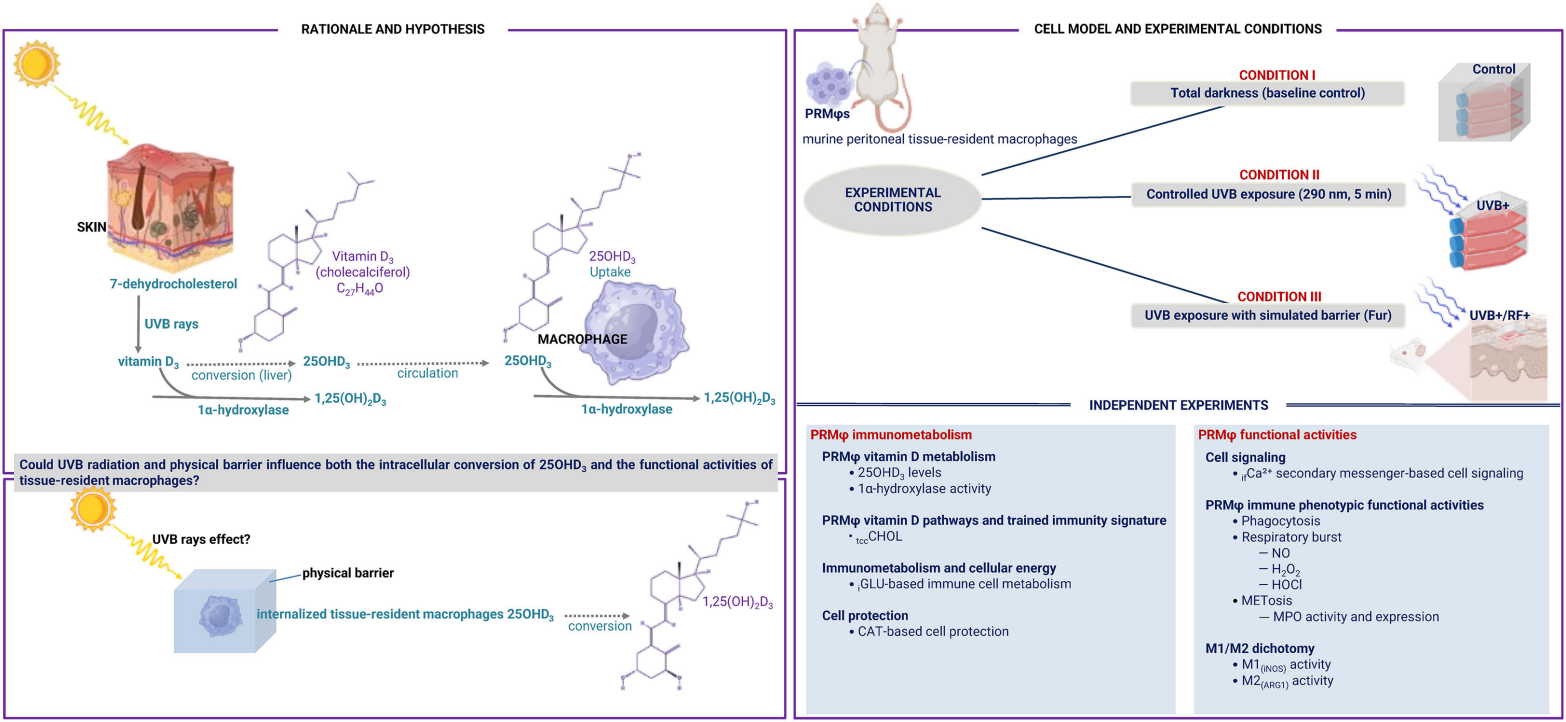
Flowchart of the current study. This study investigated the role of UVB radiation and the potential influence of passive physical barriers, such as simulated clothing, in preventing UVB exposure, on the enzymatic conversion of 25-hydroxyvitamin D3 (25(OH)D3) by 1-alpha hydroxylase, and assessed their effects on the phenotypic functional activities of tissue-resident macrophages. Experiments were conducted using murine peritoneal tissue-resident macrophages under three conditions: (i) Baseline (Control group), (ii) Controlled exposure to UVB rays (UVB+ group), and (iii) UVB exposure with a simulated physical barrier (RF+ group).

This study aims to assess the impact of UVB radiation and the potential influence of passive physical barriers on the enzymatic conversion activity of 25(OH)D_3_ and the overall functional activities of tissue-resident macrophages, as illustrated in the flowchart (**Figure 1**). Assays were conducted on whole cells, supernatants, or whole cell lysates.

### 2.1 Cell-based experimental model

In our study, we used murine peritoneal tissue-resident macrophages (PRMφs) as an experimental model, given their ability to metabolize 25(OH)D_3_ to 1,25(OH)_2_D_3_ (21), their higher stability (22), their high responsiveness to inflammatory signals, their capacity to migrate to the skin (23) along avascular routes under inflammatory conditions (24), their role in regulating inflammation (25), their ease of accessibility, their remarkable functional plasticity, and their well-established involvement in immune responses comparable to tissue-resident macrophages in other organs. Additionally, their structural and developmental similarities with other tissues, such as the skin—including comparable surface area, mesodermal origin (26), and a shared embryonic coelomic cavity—further support their relevance in studying local immune modulation. Moreover, it is not merely because normal skin lacks a sufficient number of macrophages for planned assays, but more specifically because PRMϕs are commonly used for *in vitro* assays, as they are distinguished by their maturity compared to other macrophage populations, characterized by enhanced stability in their phenotype and functionality (27).

Although the tissue was sourced from *Wistar* rats, a nocturnal species (28) that naturally displays circadian variations in metabolic and hormonal activity, the *in vitro* nature of our model allows us to minimize systemic circadian influences and focus on direct cellular responses to UVB exposure. Nonetheless, we acknowledge that macrophages possess intrinsic circadian clockworks that can remain functional even *ex vivo* (29, 30), suggesting that some degree of circadian imprinting might persist at the cellular level. While this influence is expected to be limited under controlled culture conditions, it remains an important factor to consider and explore in future *in vivo* studies.

### 2.2 PRMφs collection and preparation

The experiments were performed on primary PRMφs isolated from healthy 8-week-old female *Wistar rats* weighing 60–70 grams, with four independent repetitions (n = 12 per group), each conducted in duplicate or triplicate. To obtain PRMφs, peritoneal exudate cells (PECs) were collected from animals (31) in a sterile manner from the peritoneal cavity at different time intervals through lavage using a minimum of 10 mL sterile ice-cold phosphate-buffered saline (PBS) twice. This was achieved by intraperitoneal injection, followed by gentle abdominal massage, in accordance with established procedures as outlined elsewhere (32–35). PEC were seeded at 2 × 10^6^ cells/mL in cell culture media and incubated for 2 h at 37°C in a humidified atmosphere containing 5% CO_2_. The culture medium was changed regularly to maintain cell health. Non-adherent cells were removed by washing vigorously three times with warm RPMI-1640 medium. To activate PRMφs, adherent cells were incubated with 10 ng/mL LPS (*Escherichia coli* O26:B6, Sigma-Aldrich, St. Louis, MO, USA) in RPMI-1640 culture medium, supplemented with 10% fetal bovine serum (FBS), 100 U/mL penicillin, and 100 μg/mL streptomycin. Cell population density and viability were evaluated using the trypan blue exclusion test (TBET) with a hemocytometer under photonic microscopy (Zeiss, Jena, Germany) (36). Cell viability percentage was calculated using the standard formula: $\text{Viability }\left(\%\right)=\left(\frac{\text{Number of viable cells}}{\text{Total number of cells}}\right)\times 100$ . After the adhesion step, the purity of PRMφs was analyzed using a Floid Cell Imaging Station (Thermo Fisher Scientific, MA, USA), routinely achieving a purity greater than 90%.

### 2.3 Experimental groups and light and barrier conditions

The experiments were conducted under three conditions:

1. **Control group:** This group was used to assess baseline levels of 25(OH)D_3_, its conversion by 25-hydroxyvitamin D3-1α-hydroxylase, and overall PRMφ functional activities in the absence of light exposure.
2. **Controlled exposure to UVB rays (UVB+ group):** This group underwent a 5-min exposure to UVB radiation to mimic sunlight exposure (37), using a UV lamp calibrated to emit biologically relevant wavelengths at 290 nm.
3. **UVB exposure with a simulated physical barrier (RF+ group):** In this group, a thin layer of rat fur was placed above the cell culture system to partially mimic the passive barrier on the skin, assessing the impact of clothing-like coverage on UVB-mediated vitamin D metabolism and its effects on macrophage activity.

### 2.4 Rationale for the selected passive physical barrier

The use of *Wistar* rat fur was selected due to its unique physical characteristics, making it a more suitable model for our experimentation. Unlike humans, rats, as animals, do not wear clothing in their natural environment, rendering their fur a functional equivalent for protection against external elements, including UV rays. Furthermore, the choice of *Wistar* rat fur was specifically aligned with the source of the studied macrophages, which were derived from *Wistar* rats, ensuring biological consistency in the experimental setup.

Moreover, *Wistar* rat fur, due to its density and texture, could provide a relatively homogeneous and passive physical barrier that might realistically simulate skin protection against UVB rays. Unlike textiles, which vary widely in composition (e.g., synthetic *vs.* natural fibers), weave density, and industrial treatments (38–42), rat fur offers a reproducible, endogenous, and uniform material. Its fibrous structure could provide a consistent and quantifiable light-attenuating interface that partially mimics some clothing while avoiding confounding factors introduced by textile variability. This natural barrier might possess light-filtering properties akin to clothing, with the added advantage of being more easily quantifiable and manipulable in a controlled environment. Its fibrous composition also allows partial diffusion of UVB rays, which could be essential for studying the impact of reduced sun exposure on vitamin D metabolism and macrophage activity. Furthermore, the structure of the fur might replicate an effective physical barrier comparable to that of some clothing while being less susceptible to variations inherent in different fabric types, as would be the case with conventional garments. Notably, since rats (including albino *Wistar* strains) are nocturnal (43, 44) and not naturally exposed to prolonged sunlight, their fur may incidentally limit UV radiation penetration. However, this is likely a byproduct of its primary functions (e.g., insulation) rather than a specific photoprotective adaptation—similar to how human hair (which provides both physical shading and an additional effect due to pigmentation that enhances UV absorption) can passively protect against both UVB and UVA radiation (45). Taken together, these considerations underscore the relevance of using *Wistar* rat fur as a passive physical barrier in our experimental model. Its consistent structure, endogenous origin, and natural light-attenuating properties provide a biologically and mechanically appropriate means to simulate reduced exposure to environmental stressors—including UV radiation—while ensuring reproducibility and relevance to the source of the studied macrophages.

### 2.5 Immune cell lysis assay

Cells were lysed for protein, _tcc_CHOL, H_2_O_2_, and arginase activity assays. Briefly, cells were detached from culture plates, and the cell pellets were lysed using 500 μL of 0.1% Triton X-100 and incubated for 30 min. A mixture of Tris-HCl and MnCl_2_ was added to stop the reaction, and the lysate was collected (46).

### 2.6 Total protein assay

Protein concentration in the cell lysates was spectrophotometrically measured at 540 nm using a commercial kit (Thermo Fisher Scientific, Inc., Middletown, CT).

### 2.7 25(OH)D_3_ assay

Cellular vitamin D 25(OH)D_3_ levels were measured using a chemiluminescent microparticle immunoassay (CMIA) on the ARCHITECT 8200 autoanalyzer (ABBOTT^®^) at the Biochemistry Department of Tlemcen University Hospital Center. Due to the low volume of the PRMφ culture supernatant, a volume-adjusted serum with a known concentration of vitamin D was used to ensure detectability, and the vitamin D concentration in the supernatant alone was then determined by subtracting the serum concentration from the total measured concentration.

### 2.8 1α-hydroxylase activity based-25(OH)D_3_ bioconversion assay

To measure PRMφ 1α-hydroxylase activity, we incubated cell lysates with serum containing a known concentration of 25(OH)D_3_ for two distinct time periods—30 min and 2 h, using the two-point technique (47, 48). After each incubation period, we measured the concentration of 25(OH)D_3_ in the mixture. The activity of 1α-hydroxylase was calculated by determining the change in 25(OH)D_3_ concentration over time and normalizing this change by the amount of protein in the sample and the time interval, using the following formula: $\text{1}\alpha -\text{hydroxylase activity}=\frac{{\text{[25(OH)}{\text{D}}_{3}]}_{\text{initial}}{-\text{[25(OH)}{\text{D}}_{3}]}_{\text{final}}}{t\times P}$ , where *t* represents the incubation time and *P* the amount of protein in the sample (in milligrams). This approach assumes that the decrease in 25(OH)D_3_ correlates with the production of 1,25(OH)_2_D_3_, providing an estimate of the enzyme activity based on its effect on the substrate concentration.

### 2.9 PRMφs functional phenotypic activities

The functional phenotypic activity of PRMφs was assessed by phagocytosis, respiratory burst activity, and METosis-related MPO expression and activity.

#### 2.9.1 ROS-dependent NBT-based functional phagocytosis assay

The phagocytosis activity was evaluated using the nitro-blue tetrazolium (NBT, Sigma-Aldrich, Germany) assay, as previously described (49, 50). This semi-quantitative test measures the ability of phagocytic cells to reduce NBT to formazan, a black-blue crystal precipitate, reflecting the production of superoxide (O_2_^-^) during phagocytosis (51). To perform the assay, 100 µL of cell suspension was mixed with 100 µL of NBT solution, followed by incubation for 15 min at 37°C and an additional 15 min at room temperature, allowing the formation of formazan crystals within phagocytes. The extent of phagocytosis was quantified by measuring the reduction of soluble yellow NBT dye to insoluble black-blue formazan crystals within each PRMφs, using the Optika Microscope Camera and the Java-based image analysis software Fiji/ImageJ2 (NIH, USA). The level of phagocytosis was expressed as the percentage of NBT-positive cells.

#### 2.9.2 Respiratory burst assay

Oxidative/respiratory burst was performed by measuring NO production, H_2_O_2_ levels (52), and HOCl levels (53).

**a) NO assay**

NO production levels were spectrophotometrically determined on supernatants based on the sensitive colorimetric Griess reaction measuring the accumulation of oxidative metabolites (NOx, nitrite [NO_2_-] and nitrate [NO_3_-]). The assay involved the use of vanadium chloride (III) (VCIII), and Griess reagent (Sigma-Aldrich, St. Louis, MO, USA). First, 50 μL of supernatants from the PRMφs culture was seeded into 96-well microtiter plates with 50 μL VCIII (8 mg/mL) and 25 μL of Griess reagent. The absorbance was read at 540 nm using the ELISA plate reader (Biochrom Anthos 2020, Cambridge, UK). After 30 min incubation at 37°C, concentrations of NOx were determined from a linear standard curve established by 0–150 μmol/L sodium nitrite (NaNO_2_).

**b) H_2_O_2_ assay**

H_2_O_2_ levels were measured by the adapted method of Pick and Keisari. This method consists of the use of a buffered Phenol Red Solution (PRS), which contains a peroxide assay buffer (PAB) (5.0 mM K_2_HPO_4_, 1.0 mM KH_2_PO_4_, 140 mM NaCl, 0.5 mM glucose adjusted to pH 7.4), 0.28 mM (0.1 g/L) of phenol red (phenolsulfonphthalein) and 8.5 U/L (50 μg/mL) of horseradish peroxidase (HRPO, EC 1.11.1.7). The PRS solution was prepared immediately prior to the assay, by adding phenol red and HRPO to 2.1 mL of PAB at a final concentration of 0.46 mM and 0.046 U/mL, respectively. The supernatant was added to the assay mixture at a ratio of 1 to 4 and then incubated for 30 min at 37°C. To stop the reaction, 10 μL of 1 N NaOH was added. The H_2_O_2_ levels were measured spectrophotometrically at 610 nm against a blank containing buffered PRS and NaOH at the appropriate concentrations. A standard curve was prepared by the use of sequential dilutions of 30% H_2_O_2_. Concentration of H_2_O_2_ was expressed as nmol per 2 × 10^5^ cells per mL (54).

**c) HOCl assay**

The levels of HOCl were determined in cell supernatants by measuring the decomposition of H_2_O_2_ based on the *in vitro* oxidation of bromide (Br) by HOCl produced by the activated cells in the presence of chloride anion (Cl^–^) released into the extracellular medium. Therefore, 10 µL of cell supernatant in PBS was added to 10 µL of 22 mM sodium bromide (NaBr). The concentration of HOBr was determined by measuring its absorbance at 330 nm immediately after 30 min and 1 h of incubation (pH 12, ϵ_330_ = 332 M^-1^cm^-1^) (55). The level of HOCl was estimated using a standard prepared under the same conditions by adding 10 µL of 22 mM NaBr to 10 µL of 20 mM HOCl in PBS (pH 7.4) (56–58).The values were obtained using a standard curve created by serial dilutions of 5.25% (w/v) HOCl in PBS at pH 7.4.

#### 2.9.3 METosis-related MPO expression and activity assays

Myeloperoxidase (MPO, E.C.1.11.1.7) activity was assessed as a critical marker for the creation of macrophage extracellular traps (METs) (58). It was quantified using both a direct catalytic activity and immunofluorescence assays. For the catalytic activity assay, HOCl values were normalized to total protein content (59), with one unit of MPO activity defined as the amount catalyzing 1 μmol of HOCl per milligram of protein per 60 min and expressed percentage active chlorine per milligram of protein per one hour. The MPO expression was assessed by immunofluorescence assay based on the use of an anti-MPO antibody labeled with FITC (clone 5B8, Ms IgG1, BD Biosciences) for direct detection, performed on the Floid Cell Imaging Station (Thermo Fisher, MA, USA). CellProfiler 4.2.6 (Broad Institute, USA) was used to visualize the input image, delineate the contours of the MPO-marked fluorescent cell, segment the entire fluorescent cell, measure pixel intensity, and then calculate the area and perimeter of the MPO-marked cell. To ensure accurate comparisons and account for differences in cell size, normalized fluorescence intensity (NFI) was calculated as the weighted mean intensity (WMI) divided by the cell area (*µm²*), providing a measure of fluorescence intensity per unit area, and expressed as a.u./*µm²*.

### 2.10 M1/M2 dichotomy

The M1_(iNOS)_/M2_(ARG1)_ dichotomy was determined mathematically by measuring the M1-to-M2 ratio (60, 61).

**a) M1_(iNOS)_ activity assay**

iNOS (EC 1.14.13.39) activity was determined by normalizing the concentration of NO to the amount of protein per well, and the results were expressed as pmol per mg of protein per 30 min (62).

**b) M2_(ARG1)_ activity assay**

Arginase 1 activity (ARG1, EC 3.5.3.1) was assessed by a spectrophotometric assay based on evaluating the concentration of urea in PRMφs lysates after the addition of L-arginine (63). Firstly, 25 μL of cell lysates were inactivated by heating for 10 min at 56°C, then mixed with 200 μL aliquot of arginine buffer (10 mM L-arg, pH 6.4), and incubated at 37°C for 1 h. The reaction was stopped by adding acetic acid. The concentration of urea generated after arginine catabolism by arginase was measured at 600 nm (64). ARG1 activity was expressed as nanomoles of urea released per mg of proteins per 1 h (46).

### 2.11 CAT-based cell protection assay

CAT (EC 1.11.1.6) activity was determined by spectrophotometric analysis of H_2_O_2_ decomposition. Fifty microliters of cell lysates were combined with 50 µL of H_2_O_2_ and 50 µL of physiological saline. After vortexing and a 5-min incubation, 500 µL of titanium sulfate (TiSO_4_) was added and vortexed. Absorbance at 420 nm was then measured, using a blank of physiological saline and TiSO_4_.

### 2.12 Trained immunity activation-based _tcc_CHOL signature assay

_tcc_CHOL levels were measured spectrophotometrically in cell lysates by cholesterol oxidation. Free cholesterol was obtained from cholesterol esters using cholesterol ester hydrolase (EC 3.1.1.1.13), and the resulting H_2_O_2_ was detected at 505 nm with a chromogenic reagent (4-AP) in the presence of peroxidase (Trinder’s reaction). Results were expressed as µg _tcc_CHOL per mg protein.

### 2.13 _i_GLU-based immune cell metabolism assay

Intracellular glucose (_i_GLU) levels were determined spectrophotometrically in cell lysates, following supernatant removal, based on the oxidation of glucose by glucose oxidase (Gox, EC 1.1.3.4), which produces gluconic acid (C_6_H_12_O_7_) and H_2_O_2_, detected at 505 nm using Trinder’s method (65). The _i_GLU concentration was calculated by comparing the absorbance to a glucose standard curve and expressed as nanomoles per mg of protein.

### 2.14 _if_Ca^2+^ secondary messenger-based cell signaling assay

Intracellular free calcium ions (_if_Ca^2+^) levels were measured in cell culture lysates after the removal of supernatants using the ortho-cresolphthalein complexone (o-CPC) method as described in detail (63, 66, 67), and expressed as µg/mg of protein.

### 2.15 Statistical analyses

Data are presented as mean ± standard error of the mean (SEM). Statistical analyses were conducted using SPSS software version 26.0 for Windows (SPSS Inc., Chicago, IL, USA). The Kruskal-Wallis test, a non-parametric method appropriate for non-normally distributed data, was used for comparisons involving more than two groups. For normally distributed data, one-way ANOVA was applied (68). Additionally, pairwise comparisons were conducted using the Student’s *t*-test. A *p*-value of less than 0.05 was considered statistically significant.

## 3 Results

This study investigates the impact of UVB radiation and passive physical barriers on PRMφs, focusing on their effects on the enzymatic conversion of 25(OH)D_3_ into its active form, 1,25(OH)_2_D_3_, mediated by 1-alpha hydroxylase, as well as their broader influence on the global functional activities of these macrophages.

### 3.1 UVB exposure has no effect on 25(OH)D_3_ levels, but downregulates 1-alpha hydroxylase activity with the greatest impact in the simulated barrier

**Figure 2:**
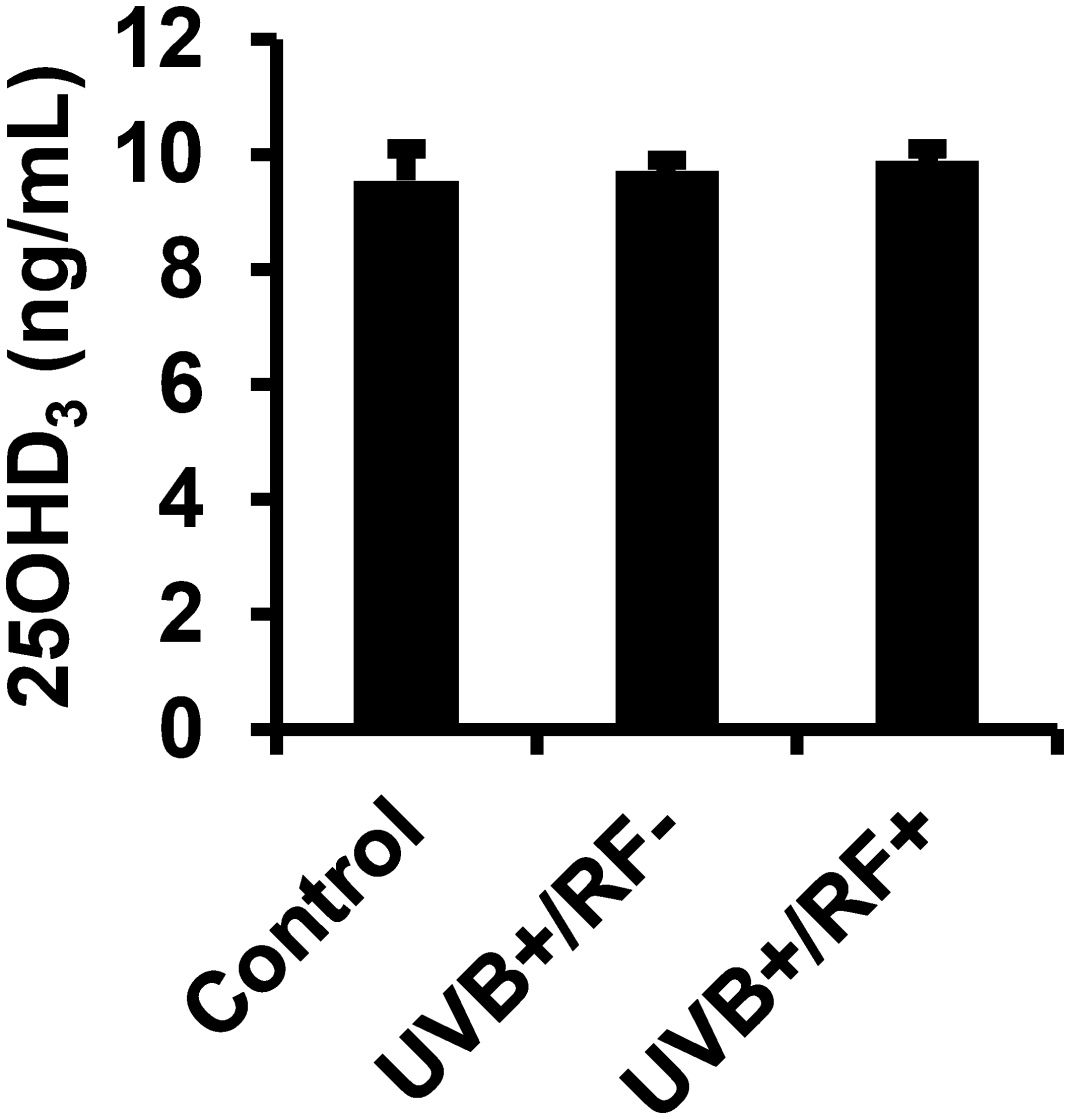
Effects of UVB radiation and passive physical barrier on 25(OH)D_3_ content of PRMφs. Experiments were conducted on murine peritoneal tissue-resident macrophages (PRMφs) under three conditions: (i) Control group, (ii) Controlled exposure to UVB rays (UVB+ group), and (iii) UVB exposure with a simulated physical barrier (RF+ group). Levels of 25(OH)D_3_ were measured using a chemiluminescent microparticle immunoassay. Data are presented as mean ± standard error of the mean (SEM) from four independent repetitions (n = 12 per group). No significant differences were observed between groups using one-way ANOVA.

**Figure 3:**
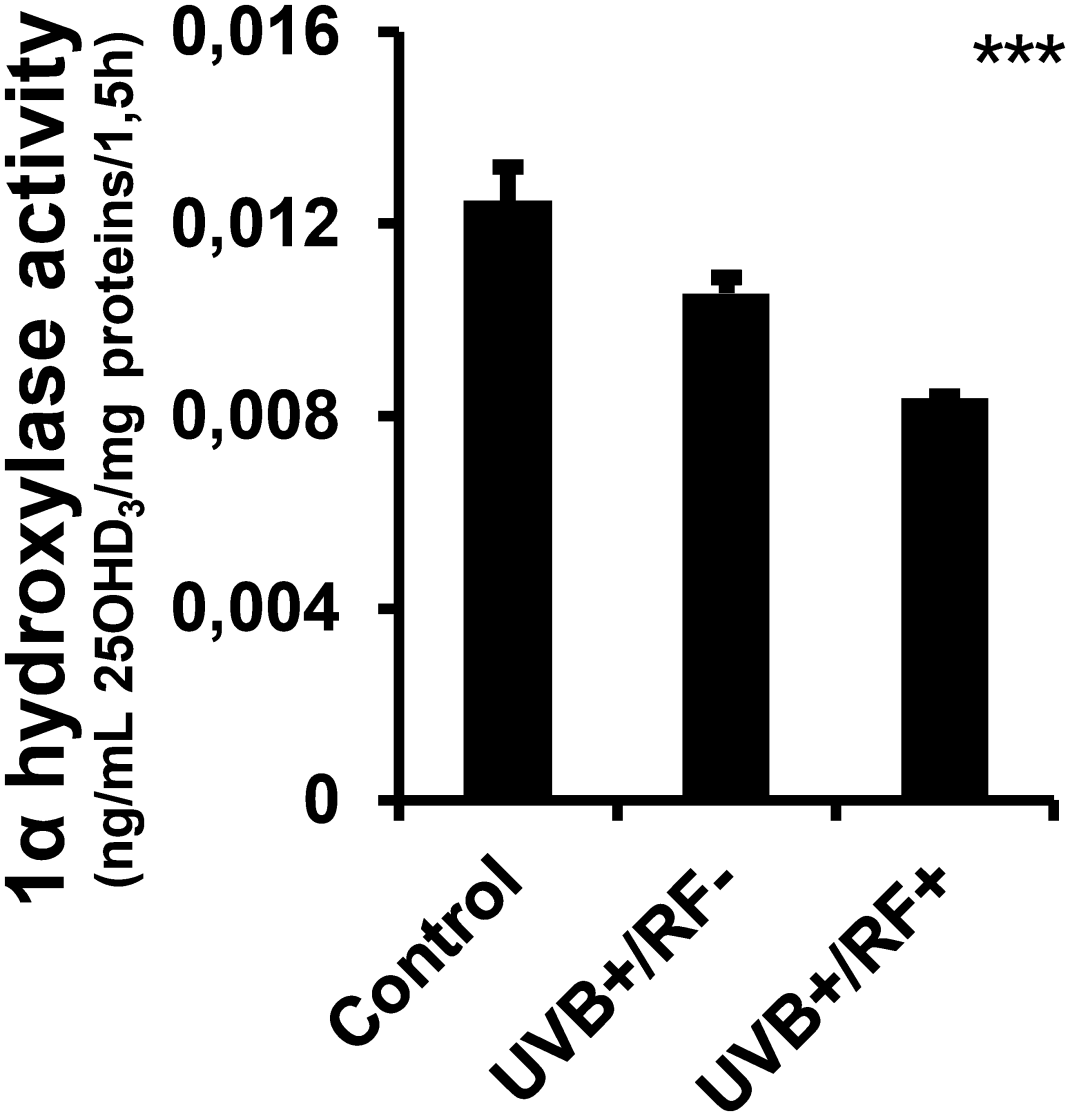
Effects of UVB radiation and passive physical barrier on 25-hydroxyvitamin D_3_-1alpha-hydroxylase of PRMφs. Experiments were conducted on murine peritoneal tissue-resident macrophages (PRMφs) under three conditions: (i) Control group, (ii) Controlled exposure to UVB rays (UVB+ group), and (iii) UVB exposure with a simulated physical barrier (RF+ group). The activity of 1α-hydroxylase assay was based on the two-point technique, determining the change in 25(OH)D_3_ concentration over time and normalizing this change by the amount of protein in the sample and the time interval. Data are presented as mean ± standard error of the mean (SEM) from four independent repetitions (n = 12 per group). Significant differences are indicated by an asterisk. Statistical analyses were performed using one-way ANOVA. ***p < 0.001.

The results presented in **Figure 2** reveal that there were no significant differences in the levels of 25(OH)D3 across the three experimental groups, irrespective of whether the PRMφs were exposed to UVB radiation alone or UVB radiation with a simulated barrier (RF+ group). In contrast, **Figure 3** highlights a significant downregulation in the activity of 1-alpha hydroxylase following UVB exposure. Notably, this downregulation was most pronounced in the simulated barrier group (UVB+/RF+) (for all comparisons, *p* < 0.001).

### 3.2 UVB increases NO production, H_2_O_2_, METosis-related MPO expression, and HOCl levels, with a moderate effect on phagocytosis, while the simulated barrier downregulates HOCl levels

**Figure 4:**
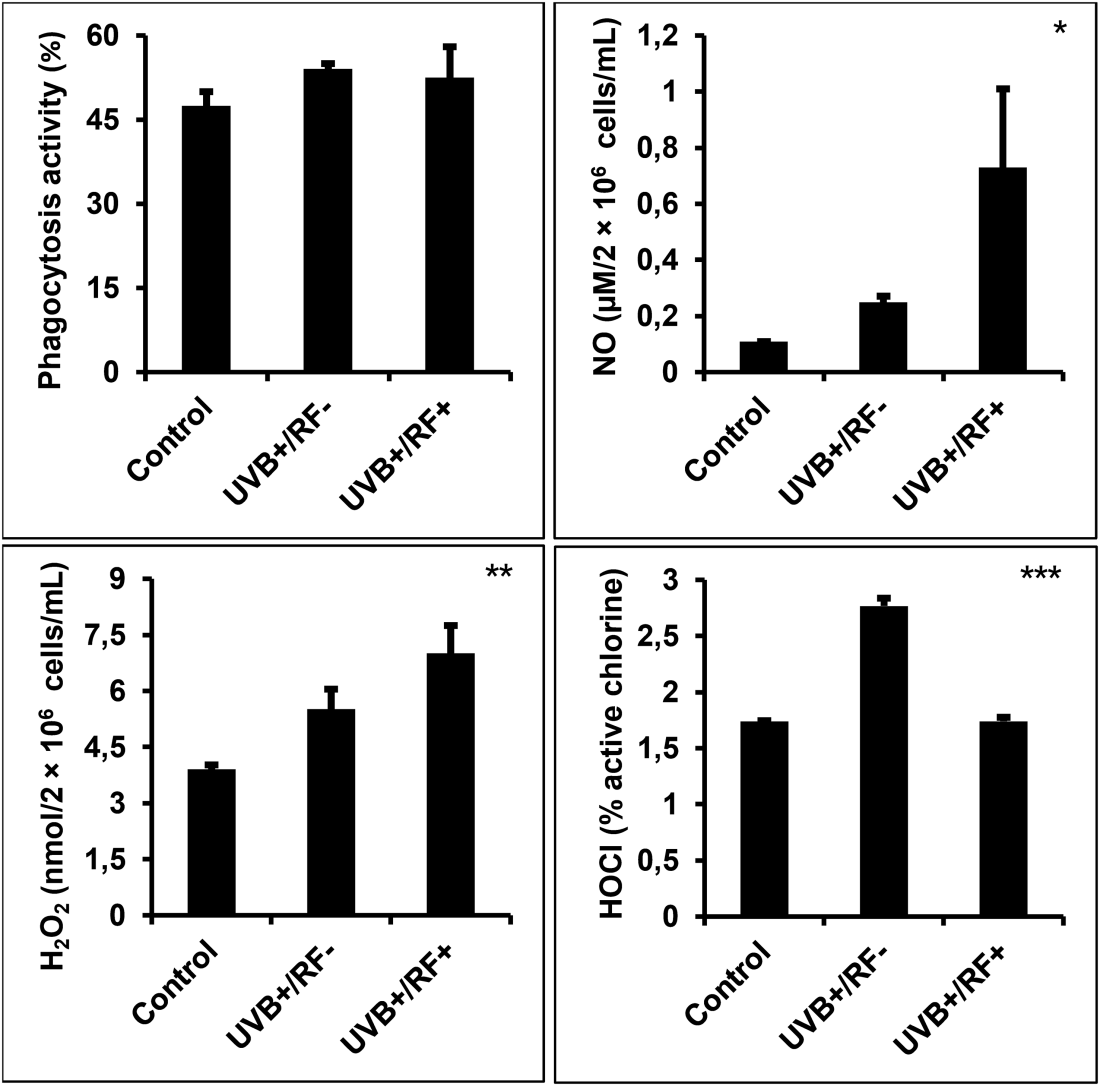
Effects of UVB radiation and passive physical barrier on phagocytosis and respiratory burst activity of PRMφs. Experiments were conducted on murine peritoneal tissue-resident macrophages (PRMφs) under three conditions: (i) Control group, (ii) Controlled exposure to UVB rays (UVB+ group), and (iii) UVB exposure with a simulated physical barrier (RF+ group). Phagocytosis activity was evaluated using the NBT method, based on its reduction to formazan by superoxide anions produced during the respiratory burst in phagocytic cells. Oxidative/respiratory burst was performed by measuring the levels of NO production, H_2_O_2_ levels, and HOCl. H_2_O_2_ levels were spectrophotometrically determined using a phenol red-based assay. NO production levels were spectrophotometrically determined using the sensitive colorimetric Griess method. HOCl levels were determined by measuring the decomposition of H_2_O_2_ through *in vitro* bromide oxidation by HOCl generated by activated cells in the presence of chloride released into the extracellular medium. Data are presented as mean ± standard error of the mean (SEM) from four independent repetitions (n = 12 per group). H_2_O_2_: hydrogen peroxide, HOCL: hypochlorous acid, NBT: nitro-blue tetrazolium, NO: nitric oxide. Significant differences are indicated by an asterisk. Statistical analyses were performed using one-way ANOVA. *p < 0.05, **p < 0.01, ***p < 0.001.

As shown in **Figure 4**, UVB exposure led to a minimal increase in phagocytic activity in PRMφs, and did not reach statistical significance. In contrast, UVB exposure—whether with or without the simulated barrier—resulted in a significant upregulation of both NO and H_2_O_2_ levels relative to the control group (*p* < 0.05 and *p* < 0.01, respectively). Moreover, the levels of HOCl were significantly upregulated in the UVB-exposed PRMφ group (*p* < 0.001); whereas, in the RF+ group, where UVB exposure was combined with a simulated barrier, HOCl levels returned to baseline levels, showing no significant difference when compared to the control group (for the comparison among the three groups using ANOVA, *p* < 0.001).

**Figure 5:**
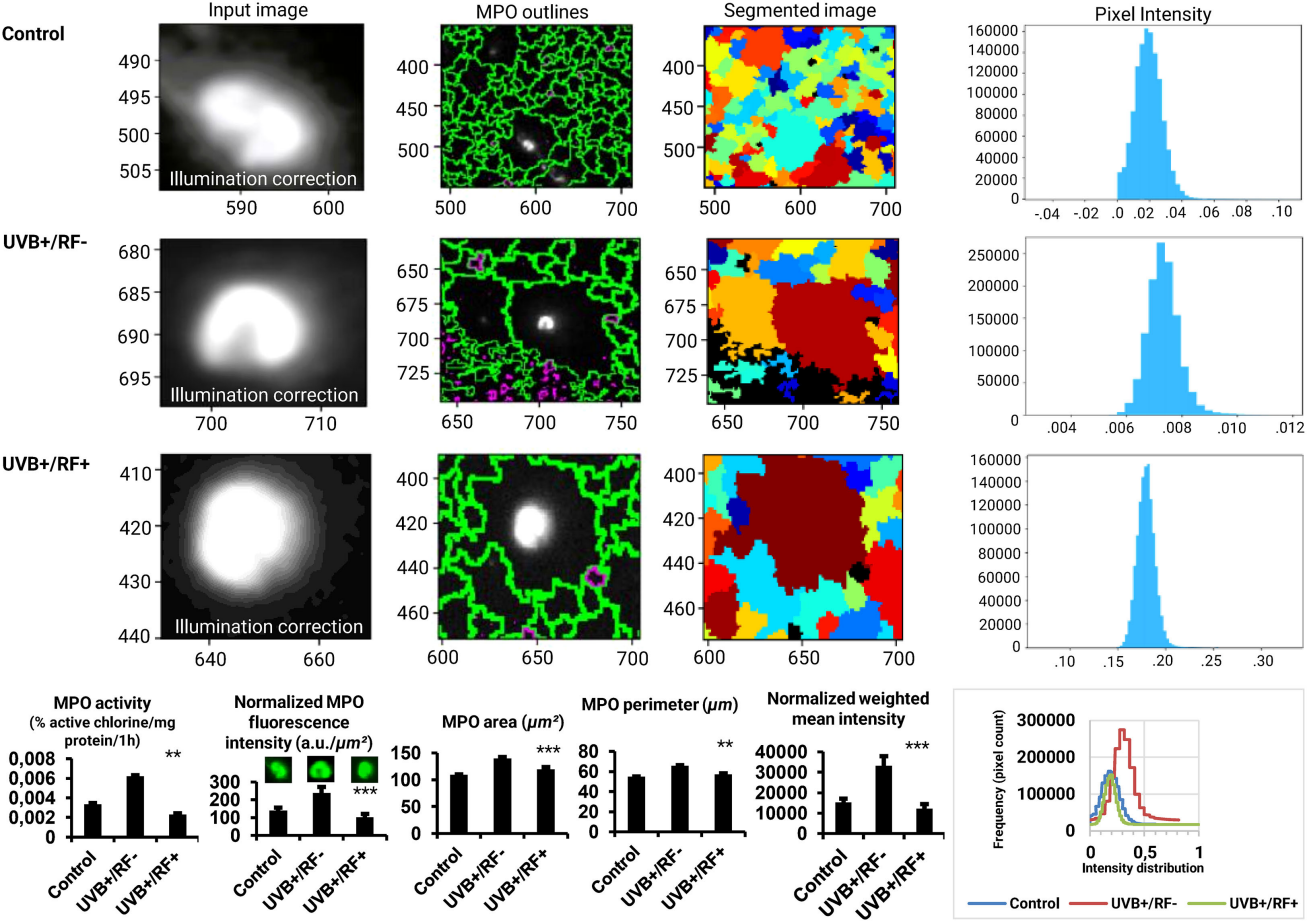
Effects of UVB radiation and passive physical barrier on MPO expression and activity of PRMφs. Experiments were conducted on murine peritoneal tissue-resident macrophages (PRMφs) under three conditions: (i) Control group, (ii) Controlled exposure to UVB rays (UVB+ group), and (iii) UVB exposure with a simulated physical barrier (RF+ group). MPO activity was quantified by directly measuring HOCl levels, normalized to total protein content, and expressed as percentage active chlorine per milligram of protein per one hour. MPO expression was assessed *via* immunofluorescence imaging, analyzed using CellProfiler software (v4.2.6, Broad Institute, USA). For imaging analysis, object dimensions were converted from pixels to micrometers (*μm*) to ensure accurate quantification. This conversion is based on an estimated image resolution of 0.58 *μm* per pixel, derived from an approximate field of view of 750 *μm* for an image width of 1296 pixels. The resolution was calculated as: $Resolution=\frac{Field\;of\;view\;width\;\left(µm\right)\;}{Image\;width\;\left(pixels\right)}=\;\frac{750\;µm\;}{1296\;pixels}\;\approx 0.5787\mu m/pixel$ . This estimation aligns with the theoretical resolution limit of the device (∼0.5 *μm*). To convert area values, the square of the resolution (0.58 *μm*/pixel)² was applied to pixel measurements, resulting in approximate areas in *μm²*. For perimeter values, a linear conversion of 0.58 *μm*/pixel was applied. Therefore, the reported values should be considered approximate. The intensity measurements were normalized using a min-max scaling approach to ensure comparability across images with differing intensity ranges. This normalization method adjusts the raw pixel intensities (I) to a scale between 0 and 1, calculated as: $\;I_{normalized}\;=\frac{I-\;I_{min}}{I_{max}-\;I_{min}},\;$ where *I_min_* and *I_max_* represent the minimum and maximum intensity values in the dataset, respectively. This process preserves the relative differences between pixel intensities while standardizing their range aligning with the quantitative expectations of the theoretical resolution limits and ensuring that the observed patterns are not influenced by varying dynamic ranges of the original images. The normalization was performed independently for each group, allowing for direct comparisons of intensity distributions across experimental conditions. Finally, the weighted mean intensity (WMI) values were calculated to account for the normalized intensity distributions across the analyzed regions of interest (ROIs). These values were derived as: $WMI=\frac{\;\sum_{i\;}w_{i}{\;}_{\times \;I_{i}}\;}{\sum_{i\;}w_{i}}$ , where I_i_ represents the intensity value of a given pixel, and W denotes the pixel area. This method ensures that the calculated intensity reflects the contribution of each pixel proportionally to its area within the ROI. Overlaid histograms representing the fluorescence intensity distribution for the three experimental groups. Intensities were normalized to ensure comparability, and the x-axis represents fluorescence intensities (a.u.), while the y-axis indicates the pixel count. For histogram data, values are presented as mean ± standard error of the mean (SEM) from four independent repetitions (n = 12 per group). HOCl: hypochlorous acid, MPO: myeloperoxidase, RF: rat fur, UVB: ultraviolet B. Significant differences are indicated by an asterisk. Statistical analyses were performed using the Kruskal-Wallis test for non-normally distributed data (area and perimeter) or one-way ANOVA for normally distributed data (normalized fluorescence intensity, normalized weighted mean intensity, and MPO activity). **p < 0.01, ***p < 0.001.

**Figure 5** further extends our analyses by specifically exploring MPO activity and expression. The analysis of MPO fluorescence intensity highlighted significant differences across the three experimental groups, underscoring the impact of UVB exposure on PRMφ activation. In contrast to the control group, where the fluorescence intensity corresponded to minimal MPO expression levels, the PRMφs group exposed to UVB displayed the highest fluorescence intensity, corresponding to the significant upregulation of MPO expression, likely triggered by UVB-induced stimulation. Lastly, in the RF+ group, where a simulated barrier limited UVB exposure, the fluorescence intensity remained similar to baseline levels. This suggests that while fluorescence intensity increased per unit area, the overall effect of UVB on METosis-related MPO activity was substantially attenuated by the simulated barrier. These findings were consistent across normalized fluorescence intensity (*p* < 0.001 by ANOVA test), area (*p* < 0.001 by Kruskal-Wallis test), perimeter (*p* < 0.01 by Kruskal-Wallis test), and normalized weighted mean intensity of MPO-marked cells (*p* < 0.001 by ANOVA test), further corroborated by the direct enzymatic activity analysis of MPO (*p* < 0.001 by ANOVA test).

### 3.3 UVB upregulates iNOS activity and shifts M1_(iNOS)_/M2_(ARG1)_ towards M1 functional phenotype

**Figure 6:**
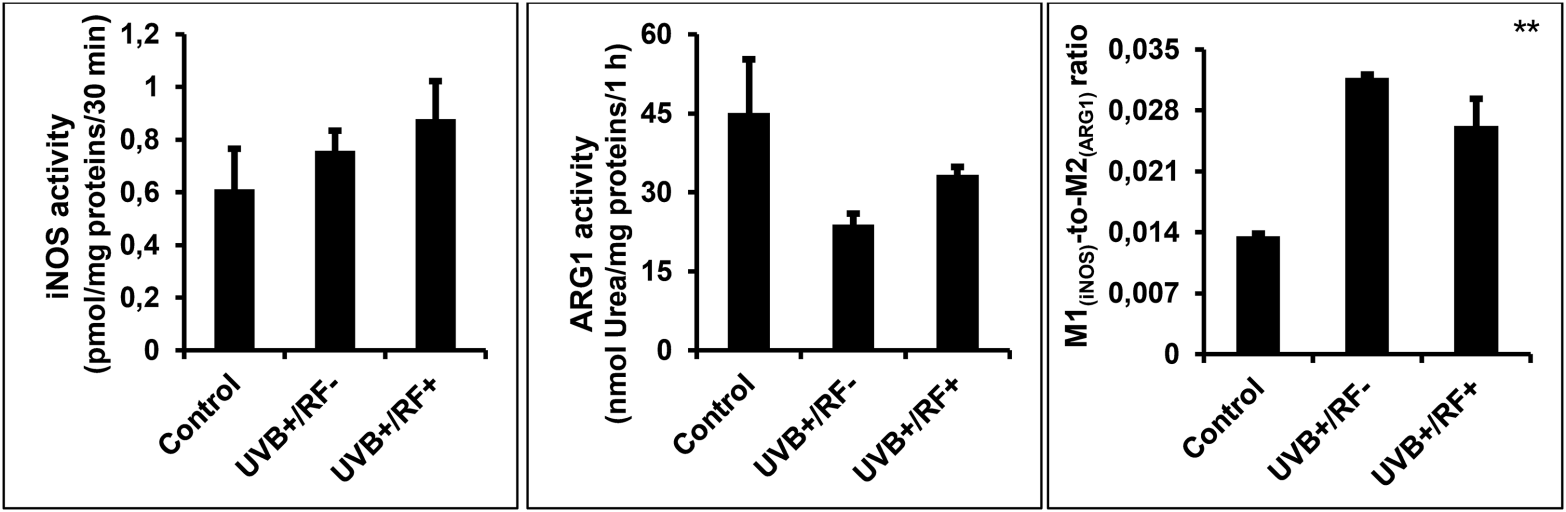
Effects of UVB radiation and passive physical barrier on the M1/M2 dichotomy. Experiments were conducted on murine peritoneal tissue-resident macrophages (PRMφs) under three conditions: (i) Control group, (ii) Controlled exposure to UVB rays (UVB+ group), and (iii) UVB exposure with a simulated physical barrier (RF+ group). The M1_(iNOS)_/M2_(ARG1)_ dichotomy was determined mathematically by measuring the M1-to-M2 ratio. M1 activity was determined by measuring iNOS (EC 1.14.13.39) activity through the quantification of NO production normalized to protein content, whereas M2 activity was assessed by measuring the amount of urea generated by ARG1 (EC 3.5.3.1). Data are presented as mean ± standard error of the mean (SEM) from four independent repetitions (n = 12 per group). iNOS: inducible nitric oxide synthase, ARG1: arginase 1. Significant differences are indicated by an asterisk. Statistical analyses were performed using one-way ANOVA. **p < 0.01.

**Figure 6** illustrates the effects of UVB exposure on the enzymatic activity of iNOS and ARG1 in PRMφs, revealing distinct modulatory patterns. Unlike iNOS activity, ARG1 activity was significantly downregulated in PRMφs exposed to UVB radiation (*p* < 0.01). The passive physical barrier similarly reduced ARG1 activity, though this effect was less pronounced compared to UVB exposure (*p* < 0.05). In contrast, neither UVB radiation nor the physical barrier significantly altered iNOS activity. Finally, both the UVB-exposed group and the RF+ group exhibited a significant increase in the M1_(iNOS)_-to-M2_(ARG1)_ ratio, indicating a shift toward a more M1-like phenotype (*p* < 0.01 by ANOVA test).

### 3.4 UVB downregulates CAT activity, specifically in simulated barrier condition

**Figure 7:**
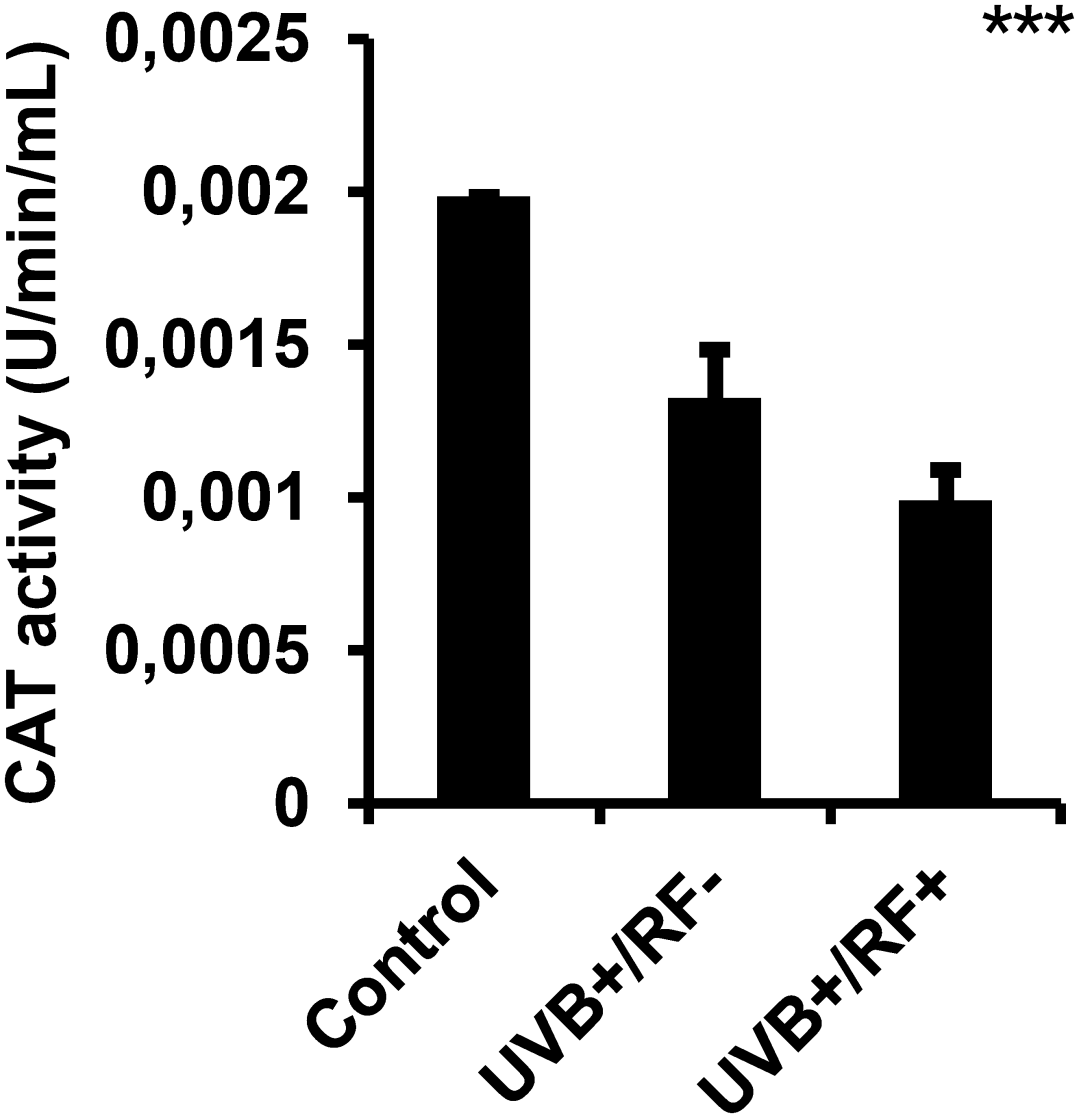
Effects of UVB radiation and passive physical barrier on catalase-based cell protection activity of PRMφs. Experiments were conducted on murine peritoneal tissue-resident macrophages (PRMφs) under three conditions: (i) Control group, (ii) Controlled exposure to UVB rays (UVB+ group), and (iii) UVB exposure with a simulated physical barrier (RF+ group). CAT (EC 1.11.1.6)-based cell protection activity was spectrophotometrically assessed by measuring H_2_O_2_ decomposition through titanium sulfate-based detection. Data are presented as mean ± standard error of the mean (SEM) from four independent repetitions (n = 12 per group). CAT: catalase activity, H_2_O_2_: hydrogen peroxide. Significant differences are indicated by an asterisk. Statistical analyses were performed using one-way ANOVA. ***p < 0.001.

As shown in **Figure 7**, UVB exposure induced a significant downregulation of CAT activity in PRMφs. This activity decreased even further in the PRMφ group with the simulated barrier (RF+ group) compared to the control group. For multiple comparisons using ANOVA test, *p*-value was less than 0.001.

### 3.5 Simulated barrier attenuates the UVB-induced increase in _tcc_CHOL

**Figure 8:**
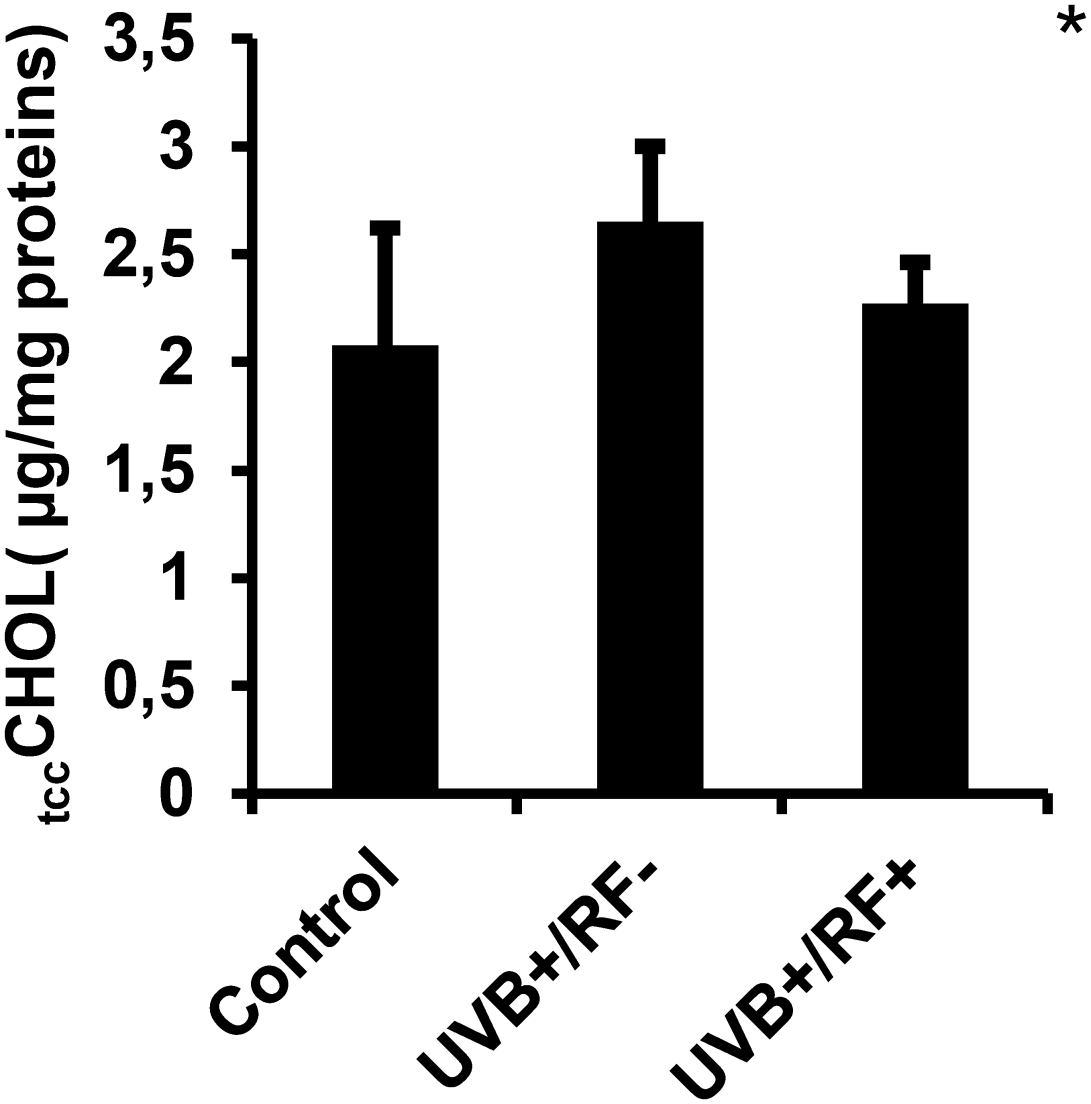
Effects of UVB radiation and passive physical barrier on trained immunity activation-based _tcc_CHOL signature of PRMφs. Experiments were conducted on murine peritoneal tissue-resident macrophages (PRMφs) under three conditions: (i) Control group, (ii) Controlled exposure to UVB rays (UVB+ group), and (iii) UVB exposure with a simulated physical barrier (RF+ group). Trained immunity activation-based _tcc_CHOL signature was measured spectrophotometrically through Trinder’s reaction. Data are presented as mean ± standard error of the mean (SEM) from four independent repetitions (n = 12 per group). _tcc_CHOL: total cellular cholesterol content. Significant differences are indicated by an asterisk. Statistical analyses were performed using one-way ANOVA. *p < 0.05.

As shown in **Figure 8**, _tcc_CHOL levels were upregulated in the UVB-exposed PRMφ group compared to the control PRMφ group, although the difference was not statistically significant. However, in the RF+ group, where UVB exposure was combined with a simulated barrier (rat fur), _tcc_CHOL levels returned to near baseline values. For all comparisons between the three groups using the Kruskal-Wallis test, the *p*-value was less than 0.05.

### 3.6 UVB decreases _i_GLU levels with a more pronounced effect under the simulated barrier condition

**Figure 9:**
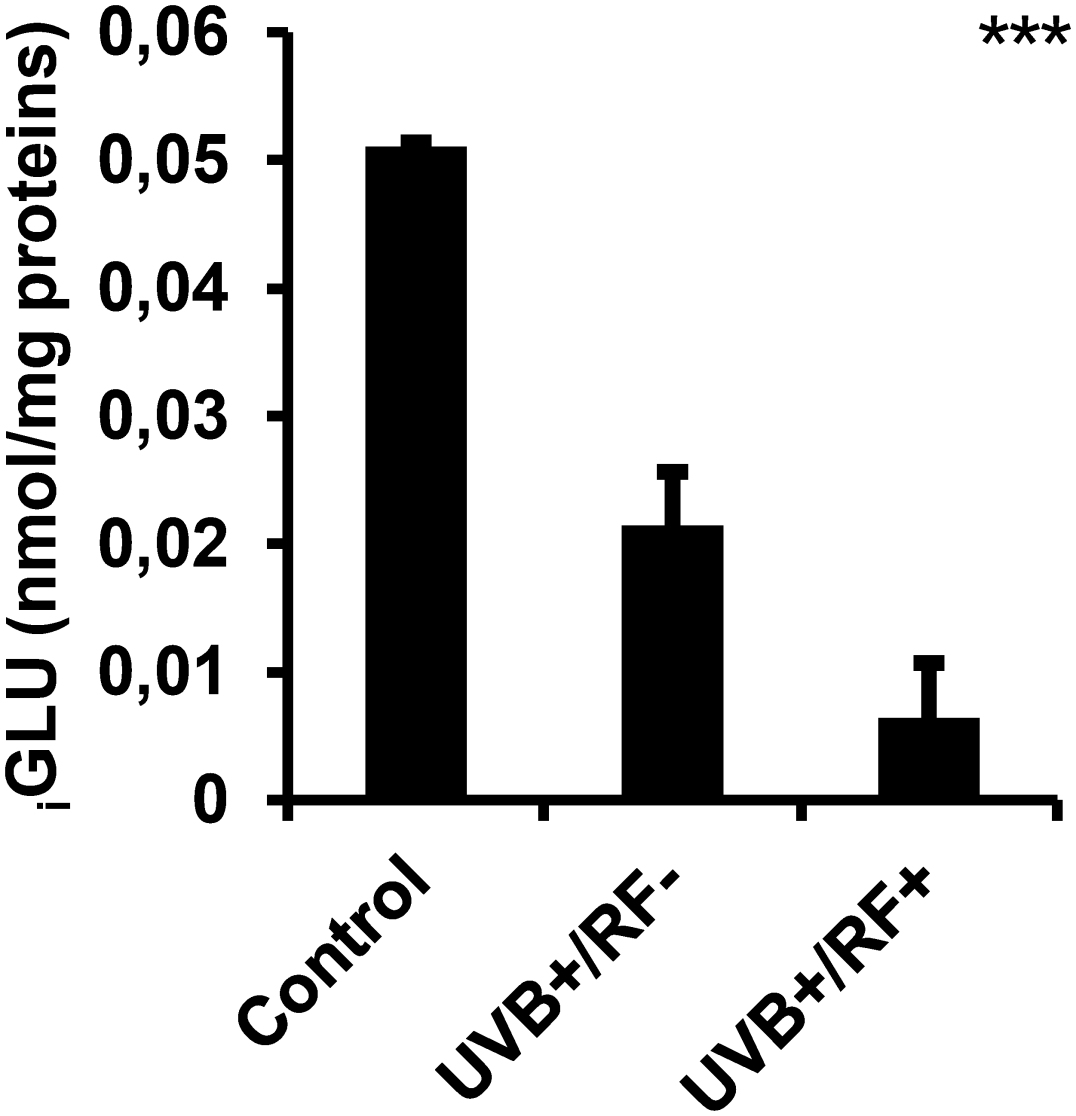
Effects of UVB radiation and passive physical barrier on _i_GLU-based immune cell metabolism of PRMφs. Experiments were conducted on murine peritoneal tissue-resident macrophages (PRMφs) under three conditions: (i) Control group, (ii) Controlled exposure to UVB rays (UVB+ group), and (iii) UVB exposure with a simulated physical barrier (RF+ group). _i_GLU-based immune cell metabolism was assessed spectrophotometrically by measuring H_2_O_2_ produced during glucose oxidation by glucose oxidase. Data are presented as mean ± standard error of the mean (SEM) from four independent repetitions (n = 12 per group). _i_GLU: intracellular glucose. Significant differences are indicated by an asterisk. Statistical analyses were performed using one-way ANOVA. ***p < 0.001.

**Figure 9** illustrates that UVB radiation, regardless of whether combined with the simulated barrier (UVB+/RF+ group) or not (UVB+/RF- group), led to a significant downregulation in _i_GLU levels compared to the control PRMφ group. Notably, the decrease in _i_GLU levels was more pronounced in the UVB+/RF+ group (*p*-value was less than 0.001 using ANOVA test), suggesting that the presence of the simulated barrier may enhance or reinforce this effect.

### 3.7 UVB exposure alone does not alter _if_Ca^2+^ levels, but a mild increase is observed with the simulated barrier

**Figure 10:**
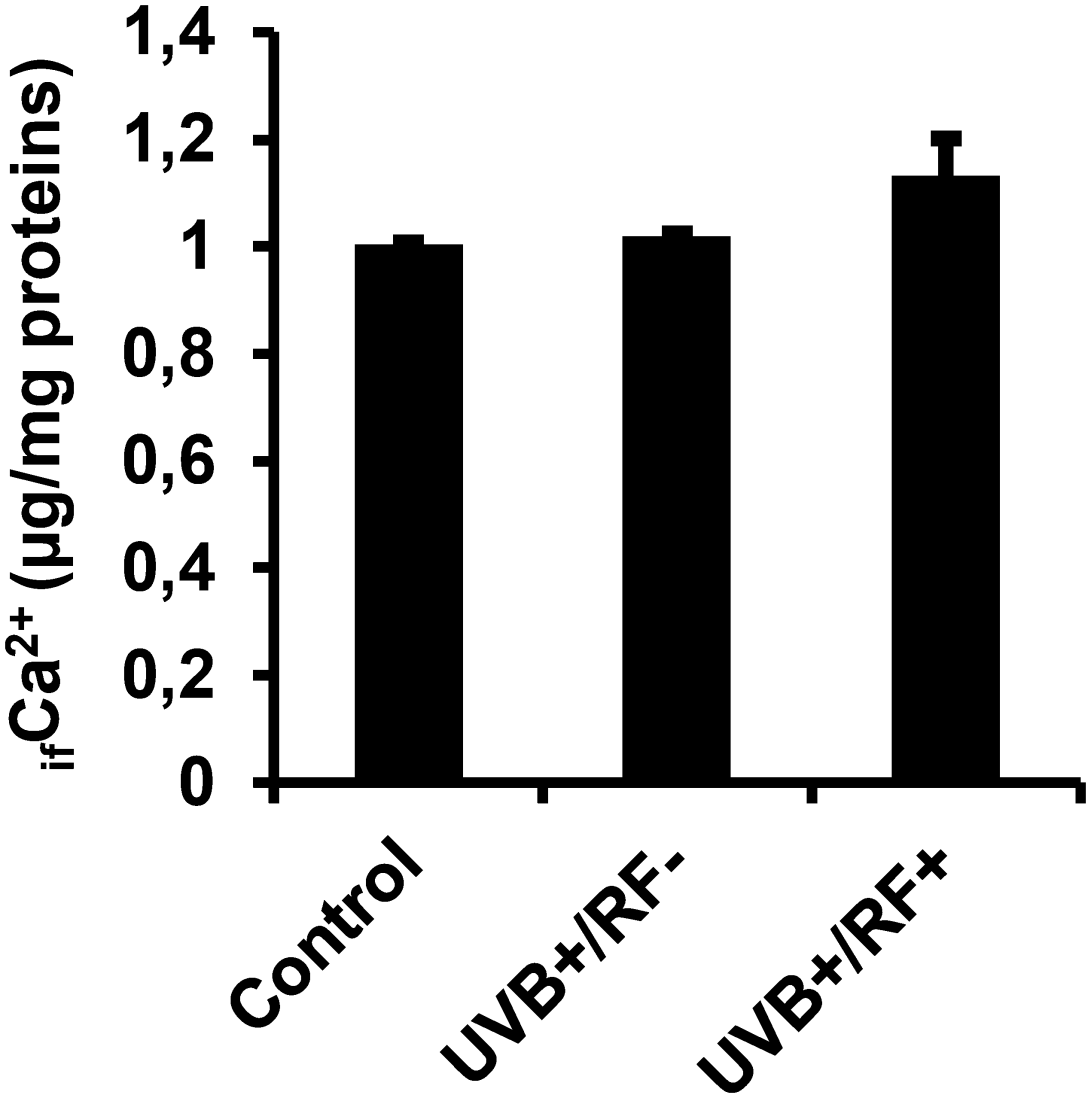
Effects of UVB radiation and passive physical barrier on free Ca^2+^ levels in PRMφs. Experiments were conducted on murine peritoneal tissue-resident macrophages (PRMφs) under three conditions: (i) Control group, (ii) Controlled exposure to UVB rays (UVB+ group), and (iii) UVB exposure with a simulated physical barrier (RF+ group). _if_Ca^2+^ levels were assessed using the ortho-cresolphthalein complexone (oCPC) method. Data are presented as mean ± standard error of the mean (SEM) from four independent repetitions (n = 12 per group). No significant differences were observed between groups using one-way ANOVA. _if_Ca^2+^, intracellular free calcium ions.

As shown in **Figure 10**, there were no significant differences in _if_Ca^2+^ levels among the three experimental groups. However, the UVB-exposed PRMφ group with the simulated barrier (UVB+/RF+) showed an upregulation in _if_Ca^2+^ levels, although this increase was not statistically significant, suggesting a subtle enhancement of calcium signaling by the simulated barrier.

## 4 Discussion

Tissue macrophages act as immune sentinels due to their strategic positioning and ability to initiate and modulate immune responses during infection or injury, while maintaining tissue homeostasis (69–71). Among these, peritoneal macrophages are well-characterized in terms of development, biology, and inflammation-related responses (72–76).

While UVB exposure from natural or artificial sources is known to modulate immunity locally and systemically, typically reducing cellular responses (77–79), our study is, to our knowledge, the first to examine how UVB radiation and passive physical barriers affect both intracellular 25OHD_3_ conversion and functional characteristics of tissue-resident macrophages under the studied conditions.

Although it might initially seem intuitive that introducing a passive physical barrier would simply attenuate UVB-induced inflammation by reducing exposure intensity, our results suggest a more nuanced and biologically significant phenomenon. The presence of a fur-based barrier did not merely lower UVB energy input, but qualitatively modulated key aspects of the macrophage response. Specifically, we observed changes in macrophage polarization (M1/M2), enzymatic activity (iNOS, MPO, Arg1), phagocytic function, and the expression of local 1α-hydroxylase involved in vitamin D activation. These findings indicate that the passive barrier influenced not only the total UVB dose but also its spectral and spatial properties—through wavelength-dependent filtering consistent with Beer–Lambert principles and modulation by light scattering. While the Beer–Lambert framework provides a basis for understanding absorption-driven attenuation, additional tissue-relevant factors such as anisotropy, optical heterogeneity, and multiple scattering—known to deviate from ideal Beer–Lambert conditions in biological systems—likely contribute to the altered UVB distribution and biological effects (80, 81). Such selective attenuation may result in change in signaling cascades within skin-resident immune cells.

In line with previous observations in tissue-specific immunomodulation, our results reinforce the notion that the local photophysical environment—including the presence of partial UVB barriers such as clothing or natural photoprotective substances (82–84)—can shape immunological outcomes in a non-linear, context-dependent manner. This distinction is clinically relevant: in autoimmune or chronic inflammatory diseases, a controlled modulation of UVB-induced macrophage activation could help limit deleterious inflammation, while in oncology or infectious contexts, preserving specific UVB wavelengths might enhance beneficial immune responses. Notably, this could extend to inflammatory bowel diseases (IBD), where systemic and local macrophage dysregulation is a known factor (85), and where skin-mediated photomodulation might exert systemic effects. Moreover, our findings may also be relevant in the context of skin inflammatory disorders such as psoriasis and atopic dermatitis, where dysregulated macrophage activation contributes to disease pathogenesis (86–90). By demonstrating that UVB exposure modulates tissue-resident macrophage function—both enhancing and restraining oxidative activity depending on barrier conditions—our study suggests that careful manipulation of UVB exposure could offer therapeutic avenues for these conditions. Further investigations in preclinical and clinical models are warranted to fully elucidate these translational possibilities.

### 4.1 Effects of UVB radiation and passive physical barrier on 25-hydroxyvitamin D_3_-1alpha-hydroxylase of PRMφs

Our findings show that UVB significantly downregulates 1α-hydroxylase activity. This may stem from the non-enzymatic UV-driven conversion of 7-dehydrocholesterol (7-DHC) to previtamin D_3_, bypassing enzymatic steps (91, 92). This aligns with the concept that metabolic pathways, including vitamin D conversion, adapt dynamically to physiological needs rather than being solely driven by circadian rhythms (93).

Supporting evidence also links vitamin D to sleep regulation: its receptor and metabolizing enzymes are expressed in sleep-regulatory brain regions (94), and vitamin D promotes melatonin synthesis—a hormone critical for circadian alignment and nocturnal physiological processes (95, 96). A systematic review in youth populations reported a link between vitamin D deficiency and disorders like insomnia, obstructive sleep apnea (OSA), and restless legs syndrome (RLS), highlighting its involvement in serotonergic/dopaminergic pathways vital to sleep (97).

Our data also show that the simulated rat barrier dampens UVB penetration, markedly reducing 1α-hydroxylase activity in the UVB+/Rat fur+ condition (18). This reinforces the concept that physical obstructions can limit local UVB-induced vitamin D metabolism by restricting UVB access.

### 4.2 Effects of UVB radiation and passive physical barrier on phagocytosis and respiratory burst activity of PRMφs

Our study revealed a modest increase in phagocytic activity of PRMφs upon direct UVB exposure. In contrast, previous *in vivo* studies reported impaired macrophage function following systemic UV irradiation, including reduced phagocytosis, bacterial clearance, and NO production (98, 99). These discrepancies likely stem from differences in experimental models—namely, our direct *in vitro* exposure versus whole-body UVB irradiation, which introduces systemic and indirect immune effects. By isolating macrophages *ex vivo*, our approach reveals a cell-intrinsic enhancement of phagocytic function, avoiding confounding influences from other immune or stromal cells.

Similarly, another study (99) showed diminished phagocytosis and active oxygen production in macrophages after UVB exposure, though neutrophils were unaffected. Our approach isolated the direct impact of UVB on PRMφs, excluding systemic factors or indirect effects mediated by other cell types. This may explain why we observed a cell-autonomous increase in phagocytic activity, whereas *in vivo* studies reported broader functional impairments due to whole-body irradiation. Furthermore, while our study did not assess other immune cells, including neutrophils, the differential effects of UVB across innate immune cell types suggest that tissue-resident macrophages may be particularly sensitive to UVB-induced modulation.

Differences in UVB doses and functional assays may also contribute to conflicting results in the literature. While other studies emphasized microbial killing, we focused on NBT-based assay to assess phagocytosis, highlighting UVB-induced ROS production during this process. Notably, phagocytosis in the UVB+/RF+ group remained higher than in the control but slightly lower than in the UVB+/RF– group, suggesting that the barrier dampens—but does not abolish—UVB effects.

The respiratory burst is central to macrophage antimicrobial defense, involving ROS and RNS generation. UVB exposure increased NO and H_2_O_2_ levels in PRMφs, even with partial UVB attenuation by fur, supporting its capacity to stimulate innate immune responses. Our NO data are consistent with reports of UVB-induced NO production in murine PRMφs (100) and human vitiligo skin (101), but differ from studies where therapeutic UVB inhibited NOS2 expression in keratinocytes and macrophages (102). Similarly, H_2_O_2_ production in response to UVB mirrors findings in neutrophils (103), mouse serum (104), and even algae (105), underscoring the evolutionary conservation of this oxidative stress response.

### 4.3 Effects of UVB radiation and passive physical barrier on MPO expression and activity of PRMφs

Our findings show that UVB exposure significantly enhances HOCl production in PRMφs, supporting our previous results on the respiratory burst. This increase is accompanied by higher MPO expression, suggesting activation of myeloperoxidase-driven oxidative mechanisms, potentially indicating METosis-like activity. The presence of rat fur, mimicking some clothing, partially attenuated this effect, highlighting its role as a passive UVB barrier—consistent with evidence that garments reduce UVB impact on skin and immune responses (106).

Elevated MPO activity, though crucial for microbial killing, is also implicated in chronic inflammatory diseases. For example, in atherosclerosis—a persistent immunoinflammatory condition—MPO-derived oxidants contribute to tissue damage, oxidized LDL accumulation, and lesion progression (107–111). Similarly, in Crohn’s disease, characterized by intestinal inflammation and immune dysregulation (112, 113), UVB-induced MPO activity may intensify oxidative stress, underlining the need to limit UVB exposure in such contexts.

Conversely, increased MPO activity in malignant conditions may support macrophage-mediated antitumor responses (114), with some studies linking higher MPO levels to reduced tumor growth and improved survival (115). This dual role suggests the context-dependent outcomes of UVB-induced MPO activation.

While rat fur differs from human fabric, it provides a consistent UVB-shielding surface. Its light-filtering properties parallel those of clothing, which varies in UV modulation based on material thickness, porosity, fiber composition, and moisture (116–120). The partial barrier function of fur may thus modulate UVB-induced inflammation without fully suppressing macrophage activity.

To assess the translational potential of our findings, future *in vivo* studies are needed to examine the systemic consequences of UVB-induced MPO activation and its modulation by passive physical barriers in disease settings. We acknowledge that our *ex vivo* model, while providing tight experimental control, does not fully recapitulate the complexity of organismal physiology. Extension to relevant *in vivo* models of chronic inflammation, cardiovascular disease, or cancer may reveal novel therapeutic applications for UVB and vitamin D–mediated modulation of macrophage function.

### 4.4 Effects of UVB radiation and passive physical barrier on the M1/M2 dichotomy

iNOS and ARG1 compete for L-arginine, their shared substrate, in activated macrophages. iNOS uses L-arginine to produce NO, whereas ARG1 converts it into L-ornithine and urea. Elevated ARG1 activity can therefore limit L-arginine availability for iNOS, reducing NO production (121).

Classically activated M1 macrophages are typically associated with high iNOS expression and NO production, representing a proinflammatory phenotype (122), whereas M2 macrophages display higher ARG1 activity, contributing to immunoregulation and wound healing (123). Thus, the iNOS/ARG1 balance shapes macrophage polarization and function (124, 125).

Our results confirm that UVB exposure enhances iNOS activity in PRMφs, consistent with previous studies in immune and skin cells (100, 126–128). Simultaneously, we observed decreased ARG1 activity, aligning with reports showing reduced M2-like macrophages after moderate UVB irradiation (129, 130). This shift in the iNOS/ARG1 ratio indicates a tendency toward an M1-like polarization. This is further supported by Karisola et al. (131), who demonstrated that UVB radiation promotes proinflammatory M1 macrophages. A potential mechanism for this polarization is UVB-induced cellular stress, which may activate proinflammatory signaling pathways, such as NF-κB (132). Additionally, UVB exposure has been shown to upregulate toll-like receptor (TLR) signaling, further driving M1 polarization (133–135).

Interestingly, the presence of a simulated barrier (RF+) further enhances iNOS activity, suggesting that the barrier may influence PRMφs beyond simple UV filtration, possibly through additional mechanical or environmental cues. Further studies are needed to determine whether this shift is transient or sustained and to elucidate the underlying molecular pathways.

### 4.5 Effects of UVB radiation and passive physical barrier on CAT activity in PRMφs

CAT is a key antioxidant enzyme that decomposes H_2_O_2_ into water and oxygen, shielding macrophages from oxidative damage (136). In our study, CAT activity peaked when PRMφs were fully shielded from UVB, emphasizing its frontline role in defending against oxidative stress. Conversely, direct UVB exposure markedly reduced CAT activity, even when partially filtered by the passive physical barrier, revealing the enzyme’s sensitivity to UVB-induced oxidative stress.

Beyond its role in neutralizing ROS, CAT is essential for maintaining redox homeostasis, which supports macrophage viability and function (137, 138). The observed suppression of CAT activity after UVB exposure indicates a weakened antioxidant defense, potentially impairing macrophage responsiveness and survival. Interestingly, the intermediate CAT activity observed in partially shielded cells suggests a threshold effect, where suboptimal UVB levels may not fully activate protective mechanisms yet still cause cellular damage.

These findings point to the need for further investigation into the molecular mechanisms underlying UVB-induced CAT alteration, including the potential involvement of redox-sensitive signaling pathways or epigenetic regulation. A deeper understanding of how environmental stress modulates macrophage antioxidant capacity could inform strategies for preserving immune cell function under oxidative conditions.

### 4.6 Effects of UVB radiation and passive physical barrier on trained immunity activation-based _tcc_CHOL signature of PRMφs

Our findings show a slight increase in _tcc_CHOL levels in UVB-exposed PRMφs, suggesting that UVB influences cholesterol metabolism, likely *via* oxidative stress and metabolic reprogramming. UVB-generated ROS (139–141) are known to affect enzymes like HMG-CoA reductase (142) and cholesterol efflux transporters such as ABCG1 (143), potentially disrupting cholesterol homeostasis. Interestingly, _tcc_CHOL levels returned to near baseline in the RF+ group, indicating that the passive physical barrier mitigated UVB effects.

Changes in _tcc_CHOL levels are also relevant to trained immunity—a long-lasting innate immune adaptation driven by metabolic and epigenetic reprogramming (144, 145). While moderate ROS can enhance trained immunity (146), excessive cholesterol accumulation may provoke dysfunction (147). Cholesterol and its metabolites shape key metabolic pathways and epigenetic remodeling, notably *via* LXR-alpha activation (148, 149), mTOR signaling (150, 151), and regulation of DNA methylation and histone acetylation. Additionally, cholesterol is an integral component of lipid rafts, which cluster PRRs like TLRs and potentiate immune signaling (152–154). Consequently, alterations in _tcc_CHOL levels could impact raft integrity and PRMφ responsiveness to secondary challenges—though this remains hypothetical and warrants experimental validation.

Finally, cholesterol’s role in modulating mitochondrial function and ROS production adds another layer of immune regulation (155). The normalization of _tcc_CHOL in the RF+ group suggests that passive physical barriers may help maintain a balance between the beneficial aspects of trained immunity and the potentially harmful effects of oxidative stress.

### 4.7 Effects of UVB radiation and passive physical barrier on _i_GLU-based immune cell metabolism of PRMφs

Glucose is the primary energy substrate supporting key macrophage functions (156). While its role in immune activation has long been recognized, its impact on cellular metabolism—particularly in linking energy production to immune responses—has only recently been fully appreciated. In macrophages, glucose not only fuels ATP production *via* glycolysis and oxidative phosphorylation (OXPHOS) but, also supports essential biosynthetic processes by providing NADPH for ROS generation, glycerol 3-phosphate for lipid synthesis, and ribose for RNA synthesis required for cytokine production (157–159).

In our study, UVB exposure led to a marked decrease in _i_GLU levels in PRMφs, likely reflecting an upregulation of oxidative metabolism and increased metabolic demand. This aligns with the observed rise in oxidative stress markers—including NO, H_2_O_2_, and HOCl—highlighting the interplay between energy metabolism and immune activation under photonic stress.

Interestingly, the simulated physical barrier further intensified the decrease in _i_GLU levels. Rather than mitigating the UVB effect, the passive barrier appeared to modulate glucose metabolism—possibly by altering glucose uptake, transporter expression, or by promoting alternative metabolic pathways activated under dual stress conditions. This unexpected outcome suggests that the barrier may function not only as a physical shield that limits UVB radiation exposure, but also as a biological modulator influencing cellular metabolism in tissue-resident macrophages in PRMφs, revealing a complex interaction between environmental stressors and immune cell bioenergetics. Further investigation is needed to elucidate the underlying mechanisms and to determine whether this effect is beneficial or detrimental to macrophage function and survival.

### 4.8 Effects of UVB radiation and passive physical barrier on _if_Ca^2+^ levels in PRMφs

_if_Ca^2+^ serves as a pivotal second messenger controlling various macrophage functions, including phagocytosis and oxidative burst in macrophages (160–162). In our study, UVB exposure alone did not significantly alter _if_Ca^2+^ levels in PRMφs. However, a mild, non-significant increase was observed in the UVB+/RF+ group, suggesting that partial attenuation and spectral modulation by the simulated barrier may subtly enhance calcium signaling.

This subtle enhancement may reflect wavelength-dependent changes in receptor- or ion channel–mediated calcium influx, as well as possible involvement of intracellular stores through endoplasmic reticulum channels like inositol 1,4,5-trisphosphate receptor (IP_3_R) and ryanodine receptor (RyR) (163), which are sensitive to ROS and calcium dynamics (164).

Additionally, mitochondrial calcium uptake *via* the mitochondrial calcium uniporter-associated channel (MiCa) channel may stimulate ATP production and ROS generation (165, 166). Since longer wavelengths can enhance mitochondrial ROS output (167–170), the spectral shift induced by the passive physical barrier—likely absorbing short UVB (292 nm) and transmitting longer wavelengths—may influence these interconnected pathways. These interlinked mechanisms are coherent with our findings of elevated NO and H_2_O_2_ levels, suggesting that barrier-modulated UVB exposure may fine-tune calcium signaling and oxidative stress responses in PRMφs. Future studies will be needed to clarify whether these calcium dynamics translate into functional changes under photonic stress.

### 4.9 Synthesis: integrated effects of UVB radiation and biological barrier on PRMφs function – spectral, energetic, and functional modulation

Our findings highlight the multifaceted impact of UVB radiation on PRMφ function, revealing that the presence of a biological barrier—simulated by rat fur—modulates both the intensity and quality of UVB reaching the cells. The barrier’s partial absorption of photons not only reduces UVB intensity but also shifts the average wavelength toward longer values, thereby decreasing photon energy, as predicted by Planck’s relation $E=\;\frac{h\;x\;c}{\lambda }$ , where *h* is Planck’s constant, *c* the speed of light, and *λ* the wavelength (171, 172). Lower-energy photons are less effective at exciting biomolecular electrons, such as those in proteins and lipids, which dampens cellular responses to photonic stress. According to the Grotthuss-Draper law, only absorbed photons can initiate photochemical changes (173–175), implying that reduced photon energy directly modulates PRMφ activity.

Beyond simple shielding, UVB photons interact with sensitive molecular targets like transient receptor potential (TRP) channels, rhodopsins (a member of the G protein-coupled receptor [GPCR] family), and flavins (176–179), thereby influencing intracellular signaling cascades. These effects involve complex phenomena at the crossroads of optics, quantum physics (e.g., photon scattering and interference), and molecular biology. The passive barrier’s selective absorption and scattering alter photon-cell interactions, potentially explaining how the barrier increases _if_Ca^2+^ levels while dampening the excitation of other pathways.

Overall, this integrated analysis underscores the dual importance of UVB intensity and spectral composition in shaping PRMφ functions. A deeper understanding of how passive physical barriers modulate photonic stress could inform strategies to preserve immune cell resilience under environmental stressors.

## 5 Conclusions and future prospects

Our study highlights the significant effects of UVB exposure on PRMφ, enhancing their functionality and shifting them toward a pro-inflammatory phenotype by increasing the M1_(iNOS)_-to-M2_(ARG1)_ ratio. UVB also induced metabolic adjustments, including downregulation of 1α-hydroxylase, catalase activity, and _tcc_CHOL—a well-established signature of trained immunity. Notably, UVB exposure led to a marked increase in MPO activity—indicative of METosis-like oxidative responses in PRMφs, but this effect was significantly reversed by the simulated physical barrier (rat fur), with MPO levels returning close to baseline. This underscores MPO’s dual role in inflammation: while it aids host defense by producing reactive oxidants—most notably HOCl, a potent antimicrobial—it can also cause collateral tissue damage when excessively or chronically activated. Attenuating MPO activity may therefore help reduce oxidative stress and limit inflammation; by contrast, broad or unregulated inhibition without protective mechanisms could compromise immune competence. These findings highlight the need for a targeted, context-aware approach to MPO modulation—one that maximizes therapeutic benefit while minimizing unintended harm.

These findings emphasize PRMφ’s role in extrarenal vitamin D metabolism and support the concept of tissue-resident macrophages serving as local “active reservoirs” of vitamin D. This non-circulating form of vitamin D, likely produced under UVB influence, could be directly utilized by immune cells to modulate localized immune responses. Our results highlight the need to go beyond circulating 25-hydroxyvitamin D levels as the sole indicator of vitamin D status and to consider tissue-specific dynamics, particularly in contexts where compensatory mechanisms are likely to influence vitamin D metabolism and utilization.

Future studies should focus on the role of tissue-resident macrophages as local vitamin D reservoirs, the long-term impact of UVB exposure on macrophage function, and the influence of passive physical barriers such as fabric or other UV-blocking materials. Additionally, extending these findings to disease models may uncover therapeutic opportunities for modulating macrophage function and vitamin D metabolism in immune-related conditions. Moreover, circadian rhythms should not be overlooked, as they can influence both macrophage reactivity and UVB-induced signaling. Incorporating temporal aspects into future experimental designs may offer deeper insights into the dynamics of immune modulation under photobiological conditions.

Beyond the insights gained from macrophage-specific effects, it is also necessary to consider the broader cellular context within which these interactions take place. Therefore, although our study centered on macrophage responses, it is important to recognize that UVB radiation and vitamin D signaling likely exert effects on multiple cell populations within the tissue microenvironment. In particular, epithelial and stromal cells are known to contribute to local immune regulation and may respond differentially to photobiological stimuli. Future studies should also investigate how these findings in a preclinical rat model could be translated to human health, with a particular focus on additional cell types involved in UVB- and vitamin D-mediated immune modulation.

## Data Availability

The original contributions presented in the study are included in the article/supplementary material. Further inquiries can be directed to the corresponding authors.
